# The impact of CEOs’ academic backgrounds on corporate financialization

**DOI:** 10.1371/journal.pone.0346327

**Published:** 2026-04-01

**Authors:** Bao Qi, Fan Hu, Chen Zhang

**Affiliations:** 1 School of Finance, Ningbo University of Finance and Economics, Ningbo, China; 2 School of Finance, Guangdong University of Finance & Economics, Guangzhou, China; 3 School of Economics, Xiamen University, Xiamen, China; Yamanashi Gakuin University: Yamanashi Gakuin Daigaku, JAPAN

## Abstract

While prior research has examined macroeconomic drivers of corporate financialization, little is known about how top executives’ personal traits shape this phenomenon. Drawing on imprinting theory, this study investigates whether and how CEOs’ academic backgrounds influence corporate financialization. Using data from Chinese A-share non-financial listed companies (2010–2022), we employ multiple regression analyses to test the relationships and underlying mechanisms. The results show that CEOs with academic backgrounds significantly reduce corporate financialization, particularly in non-state-owned enterprises and firms with lower analyst coverage. Further analysis using a mediating effect model indicates that managerial myopia and physical capital investment partially mediate the relationship between CEOs’ academic backgrounds and corporate financialization. These findings fill an important gap in the literature by revealing a micro-level behavioral channel that complements macro-level explanations of financialization. The study extends imprinting theory by identifying academic experience as a distinctive managerial imprint that shapes ethical and long-term financial decision-making, and it provides practical implications for executive selection, governance improvement, and sustainable corporate development in emerging markets.

## 1. Introduction

Corporate financialization refers to the growing tendency of non-financial firms to allocate resources to financial assets rather than to their core production or innovation activities [1–3]. This phenomenon has attracted wide attention because it may divert firms from productive investment, weaken employment growth, and undermine long-term economic sustainability [4–6]. In China, corporate financialization has intensified over the past decade. According to the China Stock Market & Accounting Research (CSMAR) database, the average financial assets held by non-financial firms listed on China’s A-share market surged from 492 million RMB (6.66% of total assets) in 2010 to 1.771 billion RMB (10.33% of total assets) in 2022. This rapid rise indicates that financialization has become a pervasive feature of corporate behavior, raising concerns about its potential crowding-out effects on real investment. Understanding what drives firms toward—or away from—financialization is therefore of considerable theoretical and practical importance.

Existing research has primarily examined the drivers of corporate financialization from a macro perspective, focusing on external factors such as economic policy uncertainty [7] and financial liberalization [8]. In contrast, studies exploring the micro-level determinants—especially managerial characteristics—remain limited. In essence, financialization is a strategic resource allocation choice, heavily shaped by the cognitive frameworks and value orientations of top executives. According to imprinting theory, experiences acquired during critical developmental or professional stages leave enduring imprints on individuals’ cognition and behavior [9–12]. Academic experience represents one such formative period, during which individuals engage in research or teaching within institutions that emphasize rationality, truth-seeking, and long-term value creation. Consequently, CEOs with academic backgrounds may display greater prudence and long-term orientation in resource allocation, favoring investments in innovation and productive assets. This raises an important question: does an academic background, by shaping a CEO’s values and decision-making preferences, influence a firm’s tendency toward financialization?

Compared with R&D or fixed-asset investment, financial assets offer shorter payback periods, higher liquidity, and potentially higher returns. These features make them attractive when managers face strong performance pressures or act on self-serving incentives. However, CEOs with academic backgrounds often possess stronger analytical rigor, risk awareness, and long-term strategic vision, developed through systematic research training [13,14]. They also tend to emphasize sustainable value creation and social responsibility [15]. Such traits may curb short-termism and reduce dependence on financial investments, encouraging firms to allocate resources toward real operations and innovation. Thus, academic experience may serve as an important individual attribute restraining corporate financialization. Nevertheless, systematic empirical evidence on this topic remains scarce, and the governance effect of the “academic imprint” on financialization is not yet well understood.

This issue is particularly relevant in China’s institutional context. Since the 1990s, market-oriented reforms and increasing labor mobility have prompted many researchers to leave academia for the corporate sector, creating a distinctive group of “scholar-type CEOs” [16]. In recent years, both national and local governments have introduced policies encouraging scientists and academics to join or establish enterprises. Data from the CSMAR database show that the share of listed firms led by CEOs with academic backgrounds increased from 12% in 2010 to 23% in 2022. As the central architects of corporate strategy and key shapers of organizational culture and value orientation, CEOs play a decisive role in shaping firms’ investment preferences, risk-taking behaviors, and resource allocation patterns. The rapid emergence of scholar-type CEOs thus presents a unique opportunity to explore how academic experience shapes managerial behavior and corporate strategies in emerging markets.

To address these questions, this study employs panel data from Chinese A-share non-financial listed firms from 2010 to 2022 and constructs a multivariate regression model to examine the relationship between CEOs’ academic backgrounds and corporate financialization. The results show that CEOs with academic backgrounds significantly reduce firms’ financial asset allocations. Furthermore, we investigate the moderating effects of external pressure and external supervision on this relationship. When firms face high performance pressure or weak external monitoring, CEOs are more likely to engage in short-sighted and self-serving behaviors [17,18]. We argue that the self-discipline and long-term orientation shaped by academic experience will exert a stronger governance effect under such conditions. To test this, we conduct heterogeneity analyses based on ownership type and analyst coverage. First, non-state-owned enterprises typically experience greater profit pressure, making their CEOs more prone to financial investments to improve short-term performance. Grouped regression results reveal that the inhibitory effect of CEOs’ academic background on financialization is more pronounced in non-state-owned firms, suggesting that academic imprinting reinforces managerial self-restraint under stronger external pressure. Second, analyst coverage serves as an important external governance mechanism [19,20] that can curb managerial opportunism. Our results show that when analyst coverage is low, the negative association between academic background and financialization becomes stronger, implying that managers’ inherent traits can play a substitutive governance role when formal external oversight is weak. Finally, we use a mediation model to explore the channels through which academic background influences corporate financialization. Empirical evidence confirms that academic experience reduces CEOs’ short-term orientation and increases firms’ investment in physical capital.

The innovations and contributions of this study are as follows: First, it expands the research perspective on corporate financialization from macro factors to the traits of micro-level decision-makers, revealing the governance effect of the CEO’s “academic imprint” in corporate financial decisions. While existing literature primarily explains the drivers of financialization through macro-institutional environments [7,21,22] or firm-level factors [23–25], less attention has been paid to the psychological and cognitive origins within decision-makers. Grounded in imprinting theory, this study starts with executives’ early experiences to elucidate how an academic background shapes rational thinking and a long-term orientation, thereby influencing corporate resource allocation preferences. This helps fill the gap of neglecting managerial characteristics. Second, this study deepens the application of imprinting theory in executive background research. Unlike existing studies that often focus on extreme environments like childhood poverty [26,27] or economic crises [10], this research examines the long-term impact of systematic professional experience—academic training—on decision-making styles. It enriches the empirical evidence for imprinting theory in the context of China’s transitional economy. Third, the findings reveal a complementary relationship between internal traits and external governance. The study finds that the self-restraint effect of academic CEOs is more prominent when external performance pressure is greater or when external monitoring is weaker. This suggests that a manager’s inherent traits can serve as an important supplement to formal institutions, offering new insights for improving corporate governance.

The findings have important implications for both practice and policy. For firms, they suggest that boards should consider candidates’ academic or research experience in executive selection and succession planning, particularly for companies pursuing innovation or long-term value creation. For policymakers, the results provide empirical support for initiatives encouraging academic–industry mobility, indicating that scholar-type CEOs can enhance corporate governance and help redirect resources from speculative finance toward productive activities.

The remainder of this paper is organized as follows. Section 2 presents the literature review and hypothesis development. Section 3 describes the data and research methodology. Section 4 reports the baseline empirical results. Section 5 conducts heterogeneity analyses, and Section 6 examines the underlying mechanisms. The final section provides a comprehensive discussion and concludes the paper.

## 2. Literature review and hypotheses development

### 2.1 Literature review

#### 2.1.1 Corporate financialization.

Research on corporate financialization seeks to explain the growing tendency of non-financial firms to shift resources from core productive activities toward financial asset allocation [1–3]. Existing literature has primarily evolved along two dimensions: the influence of external environmental factors and the role of internal firm characteristics.

One significant stream of research focuses on macroeconomic and institutional drivers. Scholars argue that broader environmental forces shape managerial incentives for financial investment. For instance, heightened economic policy uncertainty encourages firms to hold liquid financial assets as a hedging tool [7], while financial liberalization increases both the accessibility and attractiveness of financial market participation [8]. Furthermore, the prevailing shareholder-value orientation and periods of low profitability in the real sector are also identified as contextual pressures that push corporate strategy toward financial channels for profit generation [2,5].

A complementary strand examines firm-level drivers and emphasizes that organizational heterogeneity produces variation in financialization. Studies show that internal governance structures—such as ownership concentration and the presence of multiple large shareholders—can constrain financial investment [25]. Corporate strategy also plays a key role; for example, digital transformation [24] and engagement in corporate social responsibility [23] have been shown to influence asset allocation decisions. Underlying these findings is a fundamental resource allocation trade-off, where financial investment frequently crowds out physical capital expenditure and innovation [6].

While this body of work provides valuable insights, it largely treats the firm as a unitary actor responding to external pressures or internal conditions. This perspective overlooks the micro-foundational role of individual decision-makers—particularly the critical influence of the CEO, who holds ultimate authority over strategic resource allocation. Financialization is, at its core, a managerial choice, and such choices are filtered through the cognitive frameworks and personal inclinations of top executives.

#### 2.1.2 Imprinting theory and CEO experience.

Imprinting theory explains how individuals, organizations, or collectives acquire lasting characteristics during critical periods of heightened receptivity to environmental influences [28–30]. Originating from biological ecology and later applied to organizational studies, this theory identifies three core elements: (1) a critical period of increased susceptibility, (2) the significant impact of prevailing economic, technological, institutional, or cultural conditions during this period, and (3) the persistence of these imprints despite later environmental changes [9]. For example, organizations often retain structures and practices shaped by the economic and institutional contexts of their founding era [31,32], while individuals’ early career experiences leave lasting effects on their behaviors and performance [10,11].

Research applying this theory to CEOs typically investigates how formative experiences during such sensitive periods—whether professional, economic, or personal—shape their cognitive frameworks, skills, and ethical standards, thereby influencing corporate decision-making [11,12,27,33]. Since strategic choices are rooted in an executive’s established cognitive structures and past exposures, a CEO’s formative background inevitably directs their strategic approach and operational preferences [9]. Empirical studies have documented various types of imprints. For instance, CEOs who experienced economic recessions early in their careers often develop more conservative financial policies [10], while those with military backgrounds may instill a more disciplined and hierarchical organizational culture [12]. Other work examines imprints from childhood adversity, such as famine, linking them to distinct patterns in corporate social responsibility [27].

### 2.2 Hypothesis development

Among the various formative experiences documented in the literature, academic research involvement constitutes a particularly significant phase in a CEO’s early career. The academic environment—characterized by rigor, innovation, systematic thinking, and deep reflection [34]—creates a unique “sensitive period” that leaves lasting cognitive, capability, and moral imprints. These imprints influence a CEO’s cognition, competencies, and ethical orientations, which, in turn, shape their firm’s strategic and financial decision-making processes, particularly regarding financial investments. Specifically, the impacts of academic experiences can be categorized into three dimensions:

(1) **Cognitive Imprints.** Academic training fosters rigorous analytical thinking, enabling executives to approach problems logically and prudently [13,14]. This background instills habits of deep problem analysis, long-term perspective-taking, and consideration of developmental trends. CEOs with academic experience are thus more inclined to prioritize sustainable value creation over short-term profits. They are likely to allocate corporate resources to projects with sustainable competitive advantages and growth potential, rather than speculative financial transactions aimed at immediate gains. Such a long-term investment orientation reduces the level of corporate financialization.
(2) **Capability Imprints.** Academic backgrounds provide CEOs with strong analytical skills and in-depth professional knowledge, developed through systematic training and theoretical exploration [35]. These competencies enhance their ability to assess investment risks and returns, develop robust strategies, and identify innovative opportunities by combining theoretical insights with practical applications. According to resource allocation theory, when executives focus on innovation-driven activities, investments in other areas, including financial investments, tend to decline. This shift in priorities can suppress financialization within the firm.
(3) **Moral Imprints.** CEOs with academic backgrounds often exhibit heightened social responsibility, ethical standards, and self-discipline [13–15]. These qualities serve as intrinsic mechanisms to curb self-serving behaviors, especially in contexts with weaker external oversight. For instance, Zhang et al. (2020) found that scholar CEOs demonstrate stronger ethical standards and greater restraint, reducing excessive executive perks [36]. This moral orientation encourages academically trained CEOs to prioritize the organization’s long-term and societal interests over short-term financial gains. Given the high liquidity and speculative potential of financial assets, which are often used for earnings management and short-term profit adjustments [37], academically trained CEOs are less likely to engage in activities that could undermine the firm’s long-term stability or core business investments. Their ethical values discourage resource allocations focused solely on improving short-term financial results, thereby reducing corporate financialization.

In summary, academic experiences leave cognitive, capability, and moral imprints on CEOs, making them more inclined toward real investments rather than speculative financial asset allocations. Based on this reasoning, the following hypothesis is proposed:

**Hypothesis**: CEO academic background has a significant negative impact on financial investment behavior in non-financial firms.

## 3. Sample and methodology

### 3.1 Sample

Our sample covers A-share listed companies on the Shanghai and Shenzhen stock exchanges. The sample period spans 2010–2022, accounting for the substantial revisions to China’s accounting standards in 2007 and minimizing the influence of the 2008 global financial crisis on firms’ financial investment behavior. Given our focus on the financialization of non-financial enterprises, firms operating in the financial and real estate sectors—including securities, banking, insurance, and real estate—are excluded. Following established research practices, we further exclude firms listed for less than two years and those designated as ST (Special Treatment) or *ST during the sample period. Observations with missing data are removed, and all continuous variables are winsorized at the 1% level to mitigate the influence of outliers. The final sample comprises 22,367 firm-year observations.

All firm-level and financial data, including CEO academic background information, were obtained from the China Stock Market & Accounting Research (CSMAR) database, one of the most authoritative and comprehensive research-oriented databases in economics and finance in China.

### 3.2 Variable definitions

#### 3.2.1 The explained variable.

Following the approach outlined by Du et al. (2017) [6], this study measures the level of corporate financialization using the ratio of total financial assets to total assets. Specifically, the financialization degree (*Fin*) is calculated using the formula: *Fin* = (*Trading Financial Assets + Derivative Financial Assets + Net Amount of Financial Assets Purchased Under Resale Agreements + Net Amount of Loans and Advances Issued + Net Amount of Available-for-Sale Financial Assets + Net Amount of Held-to-Maturity Investments + Net Amount of Long-Term Equity Investments + Net Amount of Investment Properties)/ Total Assets*. This metric provides a comprehensive measure of the extent to which enterprises allocate their resources to financial assets relative to their total assets.

It is worth noting that the revised accounting standards introduced in 2018 significantly adjusted the accounting treatment for financial assets. Specifically, the category “*Held-to-Maturity Investments*” was renamed to “*Debt Investments*,” while “*Available-for-Sale Financial Assets*” was divided into “*Other Debt Investments*” and “*Other Equity Investments*.” As a result, when calculating *Fin* for periods post-2018, the terms “*Net Amount of Held-to-Maturity Investments*” and “*Net Amount of Available-for-Sale Financial Assets*” have been correspondingly updated.

Furthermore, this study adopts a definition of financial assets that differs from corporate accounting standards in two key respects. First, cash is excluded from the definition of financial assets. This exclusion is based on the rationale that enterprises primarily hold cash to support daily production and operational activities, rather than as a means of generating returns. Second, the study incorporates “*Investment Real Estate*” into the definition of financial assets. Although investment real estate is a tangible asset, its financial characteristics have become increasingly significant. Many enterprises hold investment real estate primarily for profit generation rather than operational use, thereby warranting its inclusion within the scope of financial assets.

#### 3.2.2 Explanatory variables.

Based on the research by Zhou et al. (2017) [38], if the CEO has previously worked in a university or research institution, the variable for CEO academic background, *Academic*, is assigned a value of 1; otherwise, it is assigned a value of 0.

#### 3.2.3 Control variables.

To control for other factors that may interfere with the research question, this study incorporates a comprehensive set of control variables commonly used in prior studies on corporate financialization and executive characteristics [18,24,39]. These variables capture both CEO-level and firm-level heterogeneity that could influence firms’ financial investment decisions. Specifically, the control variables include CEO gender (*Male*), CEO age (*Age*), CEO education level (*Degree*), whether the CEO also serves as chairman (*Dual*), company size (*Size*), leverage ratio (*Lev*), profitability (*ROA*), Tobin’s Q (*TobinQ*), ownership concentration (*Top1*), ownership balance (*Balance*), board size (*Board*), proportion of independent directors (*Indep*), years since the company’s listing (*Long*), and property rights nature (*SOE*).

Including these control variables helps minimize omitted variable bias and ensures that the estimated effect of CEOs’ academic backgrounds on corporate financialization is not confounded by other managerial or firm-specific characteristics. The specific definitions of each variable are detailed in Table 1.

**Table 1:** Variable definitions.

Variable	Definition
*Fin*	*Financial assets/ Total assets*, where *Financial assets* = *Trading Financial Assets + Derivative Financial Assets + Net Amount of Financial Assets Purchased Under Resale Agreements + Net Amount of Loans and Advances Issued + Net Amount of Available-for-Sale Financial Assets + Net Amount of Held-to-Maturity Investments + Net Amount of Long-Term Equity Investments + Net Amount of Investment Properties*.
*Academic*	If the CEO has previously worked in a university or research institution, *Academic* is assigned a value of 1; otherwise, it is assigned a value of 0.
*Male*	Whether the CEO is male, 1 for yes, 0 for no.
*Age*	*ln* (CEO’s age)
*Degree*	*Degree* is an ordinal variable representing the highest level of education attained by the CEO, with categories defined as follows: 1 = High school or below, 2 = Junior college (Associate degree), 3 = Bachelor’s degree, 4 = Master’s degree, and 5 = Doctoral degree. Higher values indicate higher levels of education.
*Dual*	Whether the chairman and CEO are the same person, 1 for yes, 0 for no.
*Size*	*ln* (Total assets)
*Lev*	Total liabilities/ Total assets
*ROA*	Net profit/ Total assets
*TobinQ*	Market value/ Total assets
*Top1*	The percentage of shares held by the largest shareholder.
*Balance*	The ratio of the combined shareholding percentage of the second to fifth largest shareholders to the shareholding percentage of the largest shareholder.
*Board*	*ln* (Number of board members)
*Indep*	Number of independent directors/ Number of board members
*Long*	*ln* (1 + Years since the company was established)
*SOE*	*SOE* is a binary variable indicating the ownership type of a firm, where SOE = 1 if the firm is state-owned and SOE = 0 otherwise.

### 3.3 Empirical strategy

We constructed the following multiple regression model to examine the impact of CEO academic background on the financialization of enterprises:


$$Fin_{it}=\alpha_{\text{0}}+\alpha_{\text{1}}Acdemic_{it}+{\alpha_{\text{2}}}^{\prime }Controls_{it}+Ind_{i}+Year_{it}+\varepsilon_{it}$$ (1)

In this model, *i* and *t* denote firm *i* and year *t*, respectively. The dependent variable, *Fin*_*it*_, measures the degree of financialization of a firm, while *Acdemic*_*it*_ represents the academic background of the CEO. ***Controls***_*it*_ is a vector of control variables that capture other firm-specific characteristics. *Ind*_*i*_ and *Year*_*t*_ denote industry and year fixed effects, respectively, which are included to control for unobserved heterogeneity across industries and time. Industry classifications follow the 2012 standards issued by the China Securities Regulatory Commission (CSRC). The manufacturing industry is further divided into categories at both the sector and subsector levels, while other industries are classified at the sector level only. *ε*_*it*_ represents the random error term.

If the estimated coefficient *β*_1_ < 0, it indicates that the CEO’s academic background exerts an inhibitory effect on the degree of corporate financialization.

### 3.4 Descriptive statistics

Table 2 provides the descriptive statistics for the variables used in this study. The average value of *Fin* is 0.078, indicating that, on average, A-share listed non-financial enterprises allocate 7.8% of their assets to financial instruments over the observation period. The minimum value of *Fin* is 0, while the maximum is 0.529, with a standard deviation of 0.104, reflecting considerable variation in the degree of financialization across different enterprises. The mean value of *Academic* is 0.222, indicating that approximately 22.2% of the companies in the sample have CEOs with academic experience. Descriptive statistics for other control variables align with findings from prior research and are therefore not discussed in detail here.

**Table 2:** Descriptive statistics of variables.

Variable	Observation	Mean	Std. Dev.	Min	Median	Max
*Fin*	22367	0.078	0.104	0.000	0.038	0.529
*Academic*	22367	0.222	0.416	0.000	0.000	1.000
*Male*	22367	0.926	0.262	0.000	1.000	1.000
*Age*	22367	50.385	6.664	33.000	51.000	66.000
*Degree*	22367	3.452	0.883	1.000	4.000	5.000
*Dual*	22367	0.326	0.469	0.000	0.000	1.000
*Size*	22367	22.125	1.196	20.118	21.940	26.054
*Lev*	22367	0.391	0.190	0.052	0.383	0.823
*ROA*	22367	0.043	0.057	−0.197	0.042	0.200
*TobinQ*	22367	2.079	1.252	0.853	1.679	7.969
*Top1*	22367	33.342	14.553	8.312	31.100	74.180
*Balence*	22367	0.788	0.625	0.034	0.625	2.907
*Board*	22367	2.114	0.196	1.609	2.197	2.639
*Indep*	22367	0.376	0.053	0.333	0.364	0.571
*Long*	22367	1.999	0.769	0.693	2.079	3.332
*SOE*	22367	0.276	0.447	0.000	0.000	1.000

## 4. Empirical results

### 4.1 Baseline results

Table 3 presents the regression results testing the impact of CEO academic background on corporate financial investment behavior using the full sample data based on Model (1). The first column reports the results without additional control variables, while the second column incorporates control variables.

**Table 3:** Baseline results.

	(1)	(2)
	*Fin*	*Fin*
*Academic*	−0.006**	−0.007**
	(−2.18)	(−2.58)
*Male*		−0.005
		(−0.99)
*Age*		0.000
		(0.71)
*Degree*		0.003**
		(2.38)
*Dual*		0.003
		(1.31)
*Size*		0.006***
		(3.24)
*Lev*		−0.151***
		(−14.16)
*ROA*		−0.026
		(−1.21)
*TobinQ*		0.001
		(1.03)
*Top1*		−0.000
		(−0.07)
*Balence*		−0.001
		(−0.27)
*Board*		−0.025***
		(−2.59)
*Indep*		−0.025
		(−0.84)
*Long*		0.028***
		(10.14)
*SOE*		−0.005
		(−1.09)
*Constant*	0.080***	−0.004
	(46.17)	(−0.08)
*Ind*	Yes	Yes
*Year*	Yes	Yes
*N*	22367	22367
*Adj. R* ^*2*^	0.105	0.175

Note: This table provides the results for the baseline model given by Equation (1). *, **, and *** denote statistical significance of the regression coefficients at the 10%, 5%, and 1% levels, respectively. Values in parentheses are t-values, with robust standard errors clustered at the firm level. We also calculate the Variance Inflation Factors (VIFs) for all explanatory variables. All VIF values are below 5, indicating that multicollinearity is not a concern.

As shown, the coefficient estimate for *Academic* is significantly negative at the 5% significance level in both columns. Specifically, in the first column, without control variables, the coefficient estimate for *Academic* is −0.006. This implies that employing a CEO with an academic background reduces the proportion of financial assets held by the firm by 0.6%. Given that the average financialization level in the sample is 7.8%, this reduction corresponds to an average decrease of 7.69% (−0.006/ 0.078 × 100%).

In the second column, which includes control variables, the coefficient estimate for *Academic* is −0.007. This indicates that employing a CEO with an academic background reduces the proportion of financial assets held by the firm by 0.7%. Relative to the average financialization level of 7.8%, this represents an average reduction of 8.97% (−0.007/ 0.078 × 100%).

In summary, both the statistical significance and the economic implications of the coefficient estimates for *Academic* indicate that employing CEOs with academic backgrounds can effectively curb the financial investment behavior of non-financial enterprises. These empirical results support research hypothesis.

### 4.2 Robustness checks

#### 4.2.1 Alternative measure of corporate financialization.

To enhance the robustness of our findings, we re-evaluated the degree of corporate financialization using an alternative metric inspired by Krippner (2005) [3]. This measure is based on the assumption that a firm’s profit composition reflects its strategic balance between financial and real activities. A higher proportion of income generated through financial channels indicates greater reliance on financial operations and, therefore, a higher level of financialization. Accordingly, we calculatefinancialization as the ratio of profits earnedthrough financial channels—such as investment income, gains from fair value changes, and other comprehensive income—to operating profits. To address potential bias caused by firms reporting negative operating profits, we standardize this measure using the following formula: *Fin2* = (*Financial Channel Profits – Operating Profits*)*/ |Operating Profits|.*

The regression results based on the revised measurement method for corporate financialization are presented in columns (1) and (2) of Table 4. The results indicate that, irrespective of whether additional control variables are included, the coefficient estimate for *Academic* remains significantly negative at the 1% level. These findings align with our previous results, demonstrating that the conclusions remain robust even after employing an alternative measurement method for corporate financialization.

**Table 4:** Robustness checks results: alternative measurement, fixed effects, and instrumental variables.

	(1)	(2)	(3)	(4)	(5)	(6)
	*Fin2*	*Fin2*	*Fin*	*Fin*	*Academic*	*Fin*
*Academic*	−0.101***	−0.086***	−0.006***	−0.005**		−0.032**
	(−4.87)	(−4.88)	(−2.64)	(−2.05)		(−2.39)
*Acdemic_Ind*					0.931***	
					(18.30)	
*Male*		0.022		−0.007	0.014	−0.004*
		(0.74)		(−1.31)	(1.42)	(−1.77)
*Age*		−0.000		−0.000	0.010***	0.000**
		(−0.19)		(−0.76)	(24.92)	(2.24)
*Degree*		0.026***		0.001	0.079***	0.005***
		(2.89)		(0.39)	(25.79)	(4.13)
*Dual*		−0.003		−0.001	0.139***	0.007***
		(−0.16)		(−0.32)	(22.69)	(2.90)
*Size*		0.020*		−0.010***	−0.001	0.006***
		(1.81)		(−3.44)	(−0.45)	(7.48)
*Lev*		−0.271***		−0.053***	−0.018	−0.152***
		(−4.04)		(−5.85)	(−0.97)	(−33.32)
*ROA*		−8.334***		−0.039**	−0.006	−0.027**
		(−58.78)		(−2.46)	(−0.12)	(−2.03)
*TobinQ*		0.052***		0.000	−0.001	0.001**
		(7.40)		(0.28)	(−0.28)	(2.09)
*Top1*		−0.004***		−0.000	−0.000	−0.000
		(−3.90)		(−0.83)	(−1.19)	(−0.24)
*Balence*		−0.068***		−0.007**	0.003	−0.001
		(−3.34)		(−2.18)	(0.43)	(−0.50)
*Board*		−0.003		0.002	0.058***	−0.024***
		(−0.05)		(0.27)	(3.29)	(−5.40)
*Indep*		0.349*		0.004	0.271***	−0.018
		(1.75)		(0.17)	(4.42)	(−1.16)
*Long*		0.097***		0.044***	−0.024***	0.027***
		(6.88)		(11.22)	(−5.13)	(22.60)
*SOE*		−0.015			−0.067***	−0.006***
		(−0.56)			(−9.04)	(−3.20)
*Constant*	−0.533***	−0.849***	0.079***	0.255***	−0.854***	−0.019
	(−43.33)	(−3.04)	(148.50)	(3.84)	(−10.26)	(−0.86)
*Firm*	No	No	Yes	Yes	No	No
*Ind*	Yes	Yes	No	No	Yes	Yes
*Year*	Yes	Yes	Yes	Yes	Yes	Yes
*N*	22353	22353	21851	21851	22367	22367
*Adj. R* ^*2*^	0.02	0.21	0.67	0.68	0.14	0.17

Note: This table provides the robustness test results for the baseline model. *, **, and *** denote statistical significance of the regression coefficients at the 10%, 5%, and 1% levels, respectively. Values in parentheses are t-values, with robust standard errors clustered at the firm level.

#### 4.2.2 Firm-level fixed effects model.

The relationship between CEO academic background and the financialization of non-financial firms may be affected by unobserved, time-invariant characteristics specific to each firm, potentially introducing endogeneity issues in our prior analysis. To address this concern and ensure the robustness of our findings, we employed an individual fixed effects model to further investigate this relationship. The results are presented in columns (3) and (4) of Table 4, where *Firm* represents the individual fixed effects of each firm.

It is crucial to note that the inclusion of individual fixed effects effectively captures the influences of industry fixed effects (*Ind*) and state-owned enterprise status (*SOE*), which are consequently omitted as independent controls in the regression analysis. Even with this modification, the coefficient estimate for *Academic* remains significantly negative at the 5% level or better, irrespective of the inclusion of additional control variables. These results offer additional support for the notion that appointing CEOs with academic backgrounds significantly curtails the financialization behavior of non-financial firms, thereby bolstering the robustness and validity of our earlier findings.

#### 4.2.3 Instrumental variable method.

To further address endogeneity issues and enhance the causal inference in this study, we use the average value of the academic background variable of CEOs in other firms within the same industry for a given year (*Acdemic_Ind*) as an instrumental variable. We then perform a two-stage least squares (2SLS) regression analysis. Theoretically, whether a firm hires a CEO with an academic background may be influenced by the overall trend of hiring academically experienced CEOs within the industry. However, the hiring practices of other firms in the industry are unlikely to directly impact the financial investment behavior of any specific firm, making *Acdemic_Ind* a feasible instrumental variable to some extent.

Column (5) of Table 4 shows the first-stage regression results, where the coefficient estimate for *Acdemic_Ind* is 0.931 (*t* = 18.30), significantly positive. The Kleibergen-Paap rk LM statistic is 331.720, and the Cragg-Donald Wald F statistic is 335.629, indicating that the model passes the tests for under-identification and weak instruments.

Column (6) of Table 4 provides the second-stage regression results, showing that the coefficient for *Acdemic* is −0.032, significantly negative at the 5% level. This finding reinforces the conclusion that appointing CEOs with academic backgrounds significantly reduces the financial asset allocation of firms.

#### 4.2.4 Propensity score matching method.

To address potential endogeneity issues arising from sample selection bias, we further apply the Propensity Score Matching (PSM) method. The sample is divided into two groups based on whether the CEO has an academic background, with all control variables from Model (1) included as covariates. We perform 1:1, 1:2, and 1:3 nearest neighbor matching with replacement to ensure comparability between the treated and control groups.

The Average Treatment Effect on the Treated (ATT) for firms with CEOs possessing academic backgrounds is estimated at −0.009 (*t* = −2.99), −0.008 (*t* = −3.42), and −0.009 (*t* = −3.74) for the 1:1, 1:2, and 1:3 matching methods, respectively. All estimates are significantly negative, indicating that CEOs with academic backgrounds significantly inhibit the financialization behavior of non-financial firms, consistent with our previous conclusions.

To further verify these findings, we re-run the regression analysis using the matched data. The results, presented in columns (1) to (3) of Table 5, corroborate the robustness of our conclusions. Specifically, column (1), based on 1:1 matching, yields a coefficient estimate for *Acdemic* of −0.006, significantly negative at the 5% level. Similarly, columns (2) and (3), corresponding to 1:2 and 1:3 matching, produce results consistent with column (1).

**Table 5:** Robustness checks results: propensity score matching, COVID-19 exclusion, and manufacturing samples.

	(1)	(2)	(3)	(4)	(5)	(6)	(7)
	*Fin*	*Fin*	*Fin*	*Fin*	*Fin*	*Fin*	*Fin*
*Academic*	−0.006**	−0.007**	−0.006**	−0.008**	−0.006*	−0.005*	−0.007**
	(−2.11)	(−2.28)	(−2.18)	(−2.51)	(−1.86)	(−1.67)	(−2.41)
*Male*	−0.001	0.002	0.001		−0.004		−0.006
	(−0.20)	(0.38)	(0.23)		(−0.66)		(−0.96)
*Age*	0.000	0.000	−0.000		0.000		0.000
	(0.42)	(0.02)	(−0.03)		(0.75)		(1.19)
*Degree*	0.004**	0.003*	0.003**		0.002		0.004***
	(2.07)	(1.85)	(2.01)		(1.30)		(2.69)
*Dual*	0.006*	0.006**	0.006**		0.002		0.001
	(1.80)	(2.12)	(2.06)		(0.52)		(0.32)
*Size*	0.007***	0.008***	0.007***		0.005**		0.007***
	(3.25)	(4.00)	(4.00)		(2.11)		(2.92)
*Lev*	−0.143***	−0.159***	−0.158***		−0.111***		−0.135***
	(−12.35)	(−14.50)	(−15.20)		(−7.93)		(−10.31)
*ROA*	0.006	−0.024	−0.013		−0.031		−0.009
	(0.18)	(−0.91)	(−0.52)		(−1.48)		(−0.49)
*TobinQ*	0.002	0.002	0.001		0.002		0.000
	(1.58)	(1.33)	(1.05)		(1.30)		(0.03)
*Top1*	−0.000	−0.000	−0.000		−0.000		−0.000
	(−0.27)	(−0.53)	(−0.88)		(−0.36)		(−0.60)
*Balence*	0.001	−0.002	−0.002		−0.002		0.001
	(0.20)	(−0.43)	(−0.69)		(−0.40)		(0.25)
*Board*	−0.013	−0.019*	−0.021**		−0.031**		−0.027***
	(−1.16)	(−1.84)	(−2.19)		(−2.57)		(−2.76)
*Indep*	0.001	−0.006	−0.012		−0.036		−0.025
	(0.02)	(−0.19)	(−0.37)		(−1.06)		(−0.78)
*Long*	0.022***	0.023***	0.024***		0.038***		0.021***
	(7.02)	(7.83)	(8.42)		(10.48)		(5.64)
*SOE*	0.005	0.004	0.003		−0.009*		−0.006
	(0.87)	(0.73)	(0.55)		(−1.65)		(−1.28)
*Constant*	−0.053	−0.047	−0.029	0.068***	−0.006	0.068***	−0.024
	(−1.13)	(−1.05)	(−0.68)	(32.63)	(−0.12)	(34.41)	(−0.50)
*Ind*	Yes	Yes	Yes	Yes	Yes	Yes	Yes
*Year*	Yes	Yes	Yes	Yes	Yes	Yes	Yes
*N*	8641	11128	12993	14260	14260	15830	15830
*Adj. R* ^*2*^	0.19	0.18	0.18	0.11	0.19	0.08	0.14

In summary, the application of the PSM method confirms that CEOs with academic backgrounds significantly curb the financialization of non-financial firms, further validating the robustness of our conclusions.

#### 4.2.5 Excluding the COVID-19 period.

The COVID-19 pandemic, which began in Wuhan in early 2020 and quickly spread worldwide, caused profound global social and economic disruptions, triggering the most severe recession since the Great Depression of the 1930s. The pandemic significantly influenced the investment behavior of Chinese non-financial firms, potentially altering their financialization patterns. To account for this impact, we exclude data from the pandemic period (2020–2022) and re-estimate the regression analysis.

The results are presented in columns (4) and (5) of Table 5. The coefficient estimates for *Acdemic* remain consistent with the baseline regression results shown in Table 3, with only minor variations in magnitude and significance levels. These findings suggest that the observed relationship between CEOs with academic backgrounds and the financialization behavior of non-financial firms is robust, even after excluding the potential confounding effects of the pandemic period.

#### 4.2.6 Robustness check using manufacturing firms only.

Given that the manufacturing sector represents the core and backbone of the real economy, we conduct a separate analysis focusing exclusively on manufacturing firms by excluding non-manufacturing samples. The regression results are presented in columns (6) and (7) of Table 5.

The findings show that, regardless of whether additional control variables are included, the coefficient estimates for *Acdemic* remain significantly negative. This suggests that CEOs with academic backgrounds significantly reduce the financialization level of manufacturing firms, further reinforcing the robustness of the conclusions drawn in this study.

## 5. Heterogeneity analysis

### 5.1 Heterogeneous effects of CEO academic background on corporate financialization across ownership types

In China, non-state-owned enterprise (NSOE) executives face greater short-term performance pressure compared to their state-owned enterprise (SOE) counterparts. This heightened pressure often drives them to adopt myopic strategies, and financial assets—with their high liquidity and quick returns—become an effective tool for boosting short-term profits. As a result, NSOE managers are more inclined to allocate resources to financial assets.

The governance role of academic experience becomes more pronounced when external pressures are high. The cognitive, analytical, and ethical imprints shaped by academic training help broaden executives’ perspectives, enhance their ability to identify high-quality real investment opportunities, and curb self-interested behavior. Therefore, in environments with strong short-term performance incentives, such as those in NSOEs, CEOs with academic experience are better equipped to resist the temptation of financial speculation, leading to a more substantial curbing effect on corporate financialization.

To test this, we conduct subsample regressions based on ownership type. The results are reported in Columns (1) and (2) of Table 6. For SOEs, the coefficient of *Academic* is statistically insignificant. In contrast, for NSOEs, the coefficient of *Academic* is significantly negative at the 1% level, indicating that CEO academic experience significantly reduces corporate financialization in non-state-owned firms.

**Table 6:** Heterogeneity analysis.

	*Fin*			
	SOE	NSOE	High analyst coverage	Low analyst coverage
	(1)	(2)	(3)	(4)
*Academic*	0.000	−0.009***	−0.003	−0.008**
	(0.01)	(−2.90)	(−0.72)	(−2.20)
*Constant*	0.039	−0.059	−0.123**	−0.099
	(0.54)	(−1.08)	(−2.27)	(−1.58)
*Controls*	Yes	Yes	Yes	Yes
*Year*	Yes	Yes	Yes	Yes
*Ind*	Yes	Yes	Yes	Yes
*N*	6176	16191	7783	13876
*Adj. R* ^*2*^	0.23	0.17	0.17	0.19
*Chow test (p-value)*	0.024		0.031	

Furthermore, we further conduct a Chow test to formally examine whether the coefficients differ across the two subsamples. The results show that the coefficient difference is statistically significant (p-value = 0.024), confirming that the inhibitory effect of CEO academic experience on corporate financialization is stronger in NSOEs than in SOEs..

### 5.2 Heterogeneous effects of CEO academic background on corporate financialization across analyst coverage

Analyst coverage plays an important role in external corporate governance. By leveraging their expertise and analytical capabilities, financial analysts collect, process, and integrate firms’ financial and non-financial information, and transmit it to investors and other market participants. This process helps mitigate the information asymmetry between companies and investors, thereby constraining managerial opportunism and short-termism [20,40,41].

At the same time, CEOs with academic backgrounds tend to exhibit stronger self-discipline and a longer-term strategic orientation. Their rational decision-making style and ethical awareness help reduce agency conflicts and the risk of self-interested behavior. When analyst coverage is high, managers are subject to closer external scrutiny, and external governance can effectively restrain opportunistic tendencies. However, when analyst coverage is low, the level of external monitoring diminishes, and information asymmetry becomes more severe, increasing the likelihood of managerial short-term behavior. In such settings, the intrinsic discipline and prudence associated with academic experience serve as a substitute governance mechanism, preventing managers from pursuing personal gains at the expense of long-term corporate value.

Based on this reasoning, we propose the following hypothesis: the inhibitory effect of CEOs’ academic backgrounds on corporate financialization is stronger when analyst coverage is low. To examine this possibility, we divide the sample into high- and low-analyst-coverage groups according to the annual median of analyst coverage. The results are reported in Columns (3) and (4) of Table 6. The coefficient of *Academic* is insignificant in the high-coverage group but significantly negative in the low-coverage group, suggesting that CEO academic experience plays a stronger governance role when external monitoring is weaker.

We further perform a Chow test to examine whether the coefficient difference between the two groups is statistically significant. The test result (p-value = 0.031) confirms that the effect of CEO academic background on corporate financialization differs significantly across levels of analyst coverage.

## 6. Mechanism analysis

### 6.1 The mitigating effect of CEO academic background on managerial myopia

Managerial myopia, grounded in time orientation theory from social psychology [42,43], represents a cognitive bias that prioritizes short-term gains over long-term value creation. Myopic managers systematically favor investments with immediate payoffs while avoiding projects requiring substantial upfront costs, extended payback periods, or uncertain future returns.

This preference manifests prominently in corporate financialization decisions. Relative to R&D expenditures, innovation initiatives, or capital-intensive projects, financial assets offer shorter investment horizons, higher liquidity, and lower short-term risk exposure—characteristics that align with the objectives of myopic decision-makers. Consequently, firms led by short-term-oriented executives exhibit elevated financial asset holdings.

CEOs with academic backgrounds, however, demonstrate distinct behavioral patterns. Academic training emphasizes evidence-based problem-solving and systematic analysis, fostering a cognitive orientation toward long-term strategic outcomes. We posit that such executives are less susceptible to managerial myopia, as their decision-making processes internalize extended time horizons and scientific rigor. This contrast establishes a theoretical pathway: CEO Academic Experience → Managerial Myopia Reduction → Corporate Financialization Mitigation.

We test this mediation mechanism using Baron and Kenny’s (1986) [44] framework through Models (2)-(4). Managerial myopia is quantified via textual analysis of MD&A sections in annual reports, operationalized as the ratio of myopia-related keywords to total word count [45]. The three-step procedure involves:

1. Estimating Model (2): Effect of CEO academic background (*Academic*) on financialization.
2. Estimating Model (3): Effect of *Academic* on managerial myopia (*Myopia*).
3. Estimating Model (4): Joint effects of *Academic* and *Myopia* on financialization.

$$Fin_{it}=\alpha_{\text{0}}+\alpha_{\text{1}}Acdemic_{it}+{\alpha_{\text{2}}}^{\prime }Controlsit+Ind_{i}+Year_{it}+\varepsilon_{it}$$ (2)

$$Myopia_{it}=\beta_{\text{0}}+\beta_{\text{1}}Acdemic_{it}+{\beta_{\text{2}}}^{\prime }{Controls}_{it}+Ind_{i}+Year_{it}+\varepsilon_{it}$$ (3)

$$Fin_{it}=\varphi_{\text{0}}+\varphi_{\text{1}}Acdemic_{it}+\varphi_{\text{2}}Myopia_{it}+{\varphi_{\text{3}}}^{\prime }{Controls}_{it}+Ind_{i}+Year_{it}+\varepsilon_{it}$$ (4)

Mediation is established if: *α*_1_ in Model (2) and *β*_1_ in Model (3) are both significantly negative, and *φ*_2_ in Model (4) is significantly positive. Full mediation occurs if *φ*_1_ becomes insignificant in Model (4); otherwise, partial mediation is inferred. For borderline cases, we supplement with Sobel tests.

Table 7 presents the mediation analysis in Columns (1) – (3). Column (1) replicates our baseline finding: *Academic* reduces financialization (*α*_1_ = −0.007, *p* < 0.05). Column (2) reveals that academic-experienced CEOs exhibit lower myopia (*β*_1_ = −0.008, *p* < 0.01). Column (3) demonstrates both persistent direct effects of *Academic* (*φ*_1_ = −0.005, *p* < 0.1) and significant positive associations between *Myopia* and financialization (*φ*_2_ = 0.073, p < 0.05), indicating partial mediation.

**Table 7:** Mechanism analysis.

	(1)	(2)	(3)	(4)	(5)
	*Fin*	*Myopia*	*Fin*	*CapitalInv*	*Fin*
*Academic*	−0.007**	−0.008***	−0.005*	0.003**	−0.005*
	(−2.58)	(−5.13)	(−1.88)	(2.13)	(−1.80)
*Myopia*			0.073**		
			(2.13)		
*CapitalInv*					−0.230***
					(−13.11)
*Constant*	−0.004	0.070***	0.000	−0.069***	−0.008
	(−0.08)	(3.63)	(0.01)	(−4.67)	(−0.19)
*Controls*	Yes	Yes	Yes	Yes	Yes
*Ind*	Yes	Yes	Yes	Yes	Yes
*Year*	Yes	Yes	Yes	Yes	Yes
*N*	22367	22367	22367	22367	22367
*Adj. R* ^*2*^	0.18	0.17	0.18	0.15	0.18

The Sobel test confirms mediation significance (Z = −5.692, *p* < 0.01), accounting for 8% of the total effect. These results collectively support partial mediation, indicating that reduced managerial myopia constitutes one channel through which academic CEOs curtail financialization.

### 6.2 The substitution effect of physical capital investment on financialization

Academic training fosters systematic thinking and ethical responsibility, predisposing CEOs toward long-term value creation over short-term profitability. Such executives are more likely to prioritize investments in physical capital—including infrastructure, equipment, and technology—that may exhibit delayed returns but confer sustainable competitive advantages. Given firms’ resource constraints, financial and physical investments often operate as substitutes. Consequently, CEOs inclined toward physical capital investment may allocate fewer resources to financial assets, establishing a secondary pathway: CEO Academic Experience → Physical Capital Investment Increase → Corporate Financialization Reduction.

We test this mechanism using Models (5) – (7), mirroring the analytical framework applied in Section 6.1. Physical capital investment (*CapitalInv*) is measured as: *CapitalInv =* Δ *(Fixed Assets + Construction in Progress + Project Materials)/ Total Assets.*


$$Fin_{it}=\alpha_{\text{0}}+\alpha_{\text{1}}Acdemic_{it}+{\alpha_{\text{2}}}^{\prime }{Controls}_{it}+Ind_{i}+Year_{it}+\varepsilon_{it}$$ (5)

$$CapitalInv_{it}=\beta_{\text{0}}+\beta_{\text{1}}Acdemic_{it}+{\beta_{\text{2}}}^{\prime }{Controls}_{it}+Ind_{i}+Year_{it}+\varepsilon_{it}$$ (6)

$$Fin_{it}=\varphi_{\text{0}}+\varphi_{\text{1}}Acdemic_{it}+\varphi_{\text{2}}CapitalInv_{it}\text{+}{\varphi_{\text{3}}}^{\prime }{Controls}_{it}+Ind_{i}+Year_{it}+\varepsilon_{it}$$ (7)

Table 7 presents the empirical results in columns (1), (4), and (5). Column (1) reaffirms the baseline negative relationship between academic background and corporate financialization (*α*_1_ = −0.007, *p* < 0.05). Column (4) reveals a distinct pattern, showing that academically trained CEOs significantly enhance physical capital investment (*β*_1_ = 0.003, *p* < 0.05). Column (5) further establishes a mediation mechanism: the negative impact of *CapitalInv* on financialization (*φ*_2_ = −0.230, *p* < 0.01) coexists with the persistent significance of the direct effect of *Academic* (*φ*_1_ = −0.005, *p* < 0.1), statistically confirming partial mediation through the physical capital investment channel.

The Sobel test validates this mediation pathway (*Z* = −3.32, *p* < 0.01), accounting for 10% of the total effect. These findings align with prior literature on investment substitution [6].

## 7. Discussion and conclusion

The academic experience of a CEO is a crucial phase in their career, significantly influencing their cognitive structure, skills, and moral character. These factors, in turn, shape their current business management and decision-making. This study, based on imprinting theory, examines the relationship between a CEO’s academic experience and the financial investment behavior of non-financial firms. Utilizing data from Chinese A-share non-financial listed companies from 2010 to 2022, our empirical analysis reveals that CEOs with academic backgrounds significantly mitigate the financialization of non-financial enterprises. Furthermore, this study examines the heterogeneity of this effect from two perspectives: property rights nature and analyst attention. The findings indicate that the inhibitory impact of a CEO’s academic experience on corporate financialization is more pronounced in non-state-owned enterprises and firms with lower levels of analyst attention. Additionally, employing a mediation model, we explore two potential mechanisms through which a CEO’s academic background influences corporate financial asset allocation: reducing managerial myopia and fostering physical investment. The results confirm that both managerial myopia and physical investment act as partial mediators in the relationship between a CEO’s academic experience and corporate financialization.

The findings are broadly consistent with prior literature emphasizing the influence of managerial traits on corporate decision-making [11,46]. Previous research has demonstrated that CEOs’ early-life experiences—such as military service, political exposure, or economic hardship—can create lasting behavioral patterns that affect firm policies [10,12,26,47–50]. However, few studies have explicitly examined academic experience as a distinctive managerial imprint. Zhou et al. (2017) showed that academically trained managers improve corporate transparency and reduce financing costs [38], while Francis et al. (2015) and Cho et al. (2017) found that professors on boards enhance corporate ethics and governance [13,14]. Zhang et al. (2020) further revealed that scholar-type CEOs exhibit greater self-discipline in resource use [36]. Consistent with these studies, our results show that academically experienced CEOs tend to avoid speculative short-term financial activities, thereby curbing financialization. By focusing on CEOs’ academic imprints, this study extends the literature beyond macroeconomic explanations of financialization [7,21] and provides a behavioral explanation rooted in managerial cognition and ethics.

The results also contribute to imprinting theory by confirming that academic experiences represent a formative period that leaves persistent cognitive, capability, and moral imprints on individuals. These imprints shape how CEOs evaluate risks, pursue long-term objectives, and adhere to ethical standards in corporate decision-making. Compared with military or political imprints, academic imprints emphasize analytical rigor, evidence-based reasoning, and social responsibility—traits that promote sustainable rather than speculative strategies. Heterogeneity analysis further reveals that the governance effect of a CEO’s academic background is more pronounced in contexts with weaker external oversight, such as low analyst attention, or under greater performance pressure, as is often the case in non-state-owned enterprises. This suggests that the self-discipline and long-term orientation internalized via academic training can function as a vital substitute for formal institutional safeguards. In the absence of robust external governance, these academically cultivated traits help curb managerial short-termism and self-serving behavior.

From a managerial perspective, appointing CEOs with academic backgrounds can help firms maintain long-term value orientation and resist excessive financialization. Boards of directors should therefore consider academic or research experience as a valuable criterion in executive selection and succession planning. From a policy perspective, these findings support recent initiatives in China that encourage the flow of talent from academia to industry (“scholars-to-entrepreneurs”), as such mobility can enhance corporate innovation and governance quality.

Overall, this study demonstrates that academic experience constitutes a distinctive managerial imprint with significant implications for corporate financial behavior. By shifting attention from macro-level institutional factors to micro-level executive characteristics, it advances the understanding of how cognitive and moral imprints shape firm strategy. In practice, firms and policymakers alike can benefit from recognizing the strategic value of knowledge-based leadership in promoting sustainable economic development.

Nonetheless, several limitations should be acknowledged. First, the binary measure of academic background used in this study (whether the CEO has worked in a university or research institution) may not capture the depth, type, or specific field of their academic engagement. Due to data availability, we are unable to distinguish, for example, between CEOs with backgrounds in science/engineering versus those in humanities/social sciences, or to identify whether the CEO holds a professional degree such as an MBA. Future research could refine this analysis by collecting more granular data to explore how different academic disciplines or professional qualifications may differentially shape corporate financial decisions. Second, as our study focuses on Chinese listed firms, cross-country comparisons would be valuable to assess the generalizability of the findings. Further research could also examine how academic imprints interact with other personal traits—such as risk tolerance, innovation orientation—to influence broader aspects of corporate behavior and long-term performance.
